# Ayurgenomics in Anti-Viral Therapy: A Literature Synthesis

**DOI:** 10.7759/cureus.34649

**Published:** 2023-02-05

**Authors:** Shadma H Quazi, Swanand S Pathak

**Affiliations:** 1 Pharmacology, Jawaharlal Nehru Medical College, Datta Meghe Institute of Medical Sciences, Wardha, IND; 2 Pharmacology, N.K.P. Salve Institute Of Medical Sciences & Research Centre, Nagpur, IND

**Keywords:** ayurveda, p4 medicine, doshas, prakriti, ayugenomics

## Abstract

This review focuses on Ayurgenomics (AG) used in antiviral therapy. According to Ayurveda, three doshas govern Prakriti, the natural organizational structure of humans. AG is a new field in modern medicine that focuses on establishing individualized self-care. It is a modern therapeutic and preventive method that enhances a person’s mental and physical well-being. Due to the threat of emerging lethal viruses and the significant role of Ayurveda in the pandemic, modern genetics studies have emerged. Prakriti, an Ayurvedic notion that AG incorporates, correlated with three *doshas* (phenotypes) called *vata, pitta,* and *kapha*. Each *Prakriti* individual had a specific balance for each *dosha*. To date, the most recent field of AG, which aims to characterize *Prakriti* types in terms of current genetics and physiology, has provided the best definition. Four databases were searched for studies on this topic using the keywords “Ayurgenomics” and “Anti-Viral Therapy.” Four articles that demonstrated a favorable approach for the application of AG were gathered for synthesis. According to this study, employing *Adhatoda Vasica* and *Cissampelos pareira L *extracts improved the viral structure of the SAR-CoV-2 virus. Further studies involving human participants are required to rule out the positive effects of AG in real-world contexts.

## Introduction and background

According to Ayurveda, health is defined as the mental, emotional, communal, and spiritual balance. This is based on strong evidence in the literature, for example, the *panchamahabhoota* concept (the five primary aspects of space, air, fire, water, and earth) [1]. *Trigunas *(three psychological attributes of *satwa*, *rajas*, and *ramas*), the characteristics of *aatma *(the soul), combine with the *panchamahabhootas* to develop the three major biological markers, namely, *vata *(VT), *pitta *(PT), and *kapha *(KP), which are representative of typical phenotypes. These three *doshas *are thought to govern a person’s particular blend of physical, physiological, and psychological characteristics in Ayurvedic philosophy. The relative proportions of these three doses affect a person’s *Prakriti* [2].

According to Ayurveda, *Prakriti *is a person’s innate constitution that has existed from the moment of their creation. Each *dosha* is thought to control a specific physiological attribute. For instance, VT is in charge of human movement; PT determines a human’s energy, and KP informs a human’s lubrication, cohesion, and structure. Some studies indicate that all life forms and species, from an individual cell to the complete body, depend on *doshas* at every level of biological organization. The three *doshas *(phenotypes) are uniquely balanced in each individual, although some *doshas* may prevail. This fundamental structure determines the individual’s *Prakriti*, which is a result of their dominant *doshas* [3,4].

Ayurgenomics (AG) can profoundly progress preventive, predictive, personalized, and participatory (P4) medical interventions by offering thorough theoretical knowledge of a patient and highlighting the implication of the practice of historical and current means of prevention and curative approaches to strengthen the patient’s overall health and wellbeing. The best definition of *Prakriti* to date has been offered by the new field of AG, which tries to classify *Prakriti *types in the context of recent genetics and physiology [5]. This rapid synthesis was performed to assess the role of AG in the antiviral therapy approach.

## Review

Research methodology

**Figure 1:**
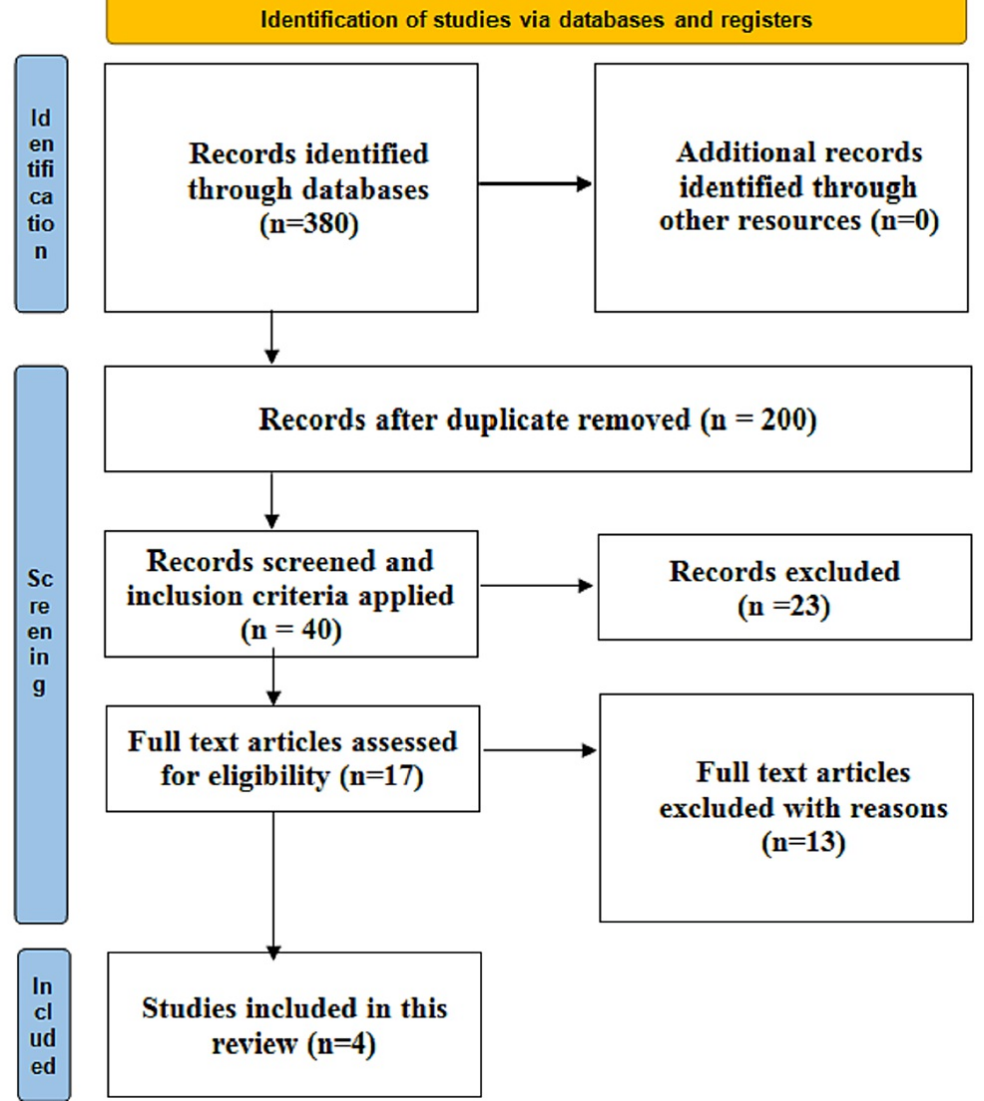
Literature search strategy.

The literature search was conducted by the principal investigator (SQ), and the identified articles were critically assessed by the second investigator (SP). The overall synthesis was performed in accordance with the Preferred Reporting Items for Systematic Review and Meta-Analyses for Rapid Review (PRISMA-RR) [6]. The search was conducted in databases such as PubMed, Google Scholar, CINAHL, and Cochrane Library using the following keywords: “Ayurgenomics” AND “Antiviral” AND “Ayurveda” “Prakriti Concept” AND “Ayurveda personalized medicine.” Articles published between 2018 and 2022, with free full texts, and in English were included for rapid synthesis. The inclusion criteria were peer-reviewed literature on AG application and implementation, intervention and clinical studies, randomized controlled trials, systematic reviews, and meta-analyses. The exclusion criteria were non-peer-reviewed literature, locked articles, abstracts, and studies published before 2018. The databases PubMed and Google Scholar showed results related to these keywords. The research strategy is illustrated in Figure 1.

Results

Only four of the 384 articles from the databases were included for rapid review, as these articles met all the inclusion criteria. Two articles were clinical trials, and two were peer-reviewed literature. The details of the reviewed studies yielded using the terms “Ayurgenomics” and “Anti-Viral Therapy,” including the type of study, author name, method, and conclusion, are presented in Table 1.

**Table 1:** Characteristics and findings of the articles extracted and included for synthesis. HIF: Hypoxia-inducible factor; CLP: Cecum ligation puncture; BALF: Bronchoalveolar lavage fluid; AV: Adhatoda Vasica; RNA: Ribonucleic acid, SAR-CoV2: Severe acute respiratory syndrome coronavirus 2; COVID-19: Coronavirus disease of 2019.

Author	Study type	Method	Conclusion
Bhat et al. [7]	Review	Literature synthesis	The suggested method of Ayurveda-based phenotypic evaluation may provide a path forward for creating personalized COVID-19 management regimens depending on variations in Prakriti, immunological performance, and medication performance. The association between Prakriti and genetic or phenotypic polymorphisms in COVID-19-susceptible and -resistant populations, however, requires clinical assessment.
Bhadresha et al. [8]	Review	Literature synthesis	The recognition of unique pathways could significantly improve our knowledge of molecular signaling between species. Cross-kingdom regulation appears to be the key to answering the unavoidable question of how these therapeutic plants influence the human transcriptome.
Haider et al. [9]	Laboratory study	The Connectivity Map and Enrichr were used for obtaining data on RNA sequencing, gene BALF 1 & 2 of SAR-CoV2 patients who received Cipa.	The antivirals such as apicidin and emetine showed positive correlations with Cipa and appeared to inhibit the coronavirus, as seen in the Vero cell culture in the whole extract.
Gheware et al. [10]	Animal experimentation	The mice were segregated into vehicle and treatment groups; the latter group was further divided based on interventional needs such as CLP, AV, and bleomycin groups.	The study reported that AV can reverse pulmonary fibrosis and reduce HIF alpha. The influence of AV on the phenotypic traits of pulmonary and systemic inflammation may also be useful in the context of the COVID-19 pandemic.

Discussion

The coronavirus disease of 2019 (COVID-19) has emerged as a global public health emergency, leading to the discovery of many antiviral drugs, vaccines, and other therapies to reduce morbidity and mortality. Severe acute respiratory syndrome coronavirus 2 (SARS-CoV-2) infection can manifest as a multitude of different illness presentations, including asymptomatic cases, moderate cases, and severe infections that can be fatal. The phenotypic characteristics of a person have a significant impact on the understanding of the inter-individual variability in illness susceptibility and prognosis [11]. To achieve objectives such as identifying major risk and illness populations and to provide tailored pharmacotherapeutic therapies, the study of these morphological changes connected to changes in genetic patterns is promising [12].

In India, various clinical trials have been conducted on different treatment modalities, especially by the six Indian systems of medicine-Ayurveda, Yoga, Naturopathy, Unani, Siddha, and Homeopathy (AYUSH)-in which herbal medicine plays a significant role in alleviating the symptoms. As traditional medicine systems have a personalized approach to the prediction of the disease, progression, and possible treatment based on their basic theories, AG integrates Ayurveda with contemporary genomes. The susceptibility to and progression of COVID-19 are thought to be correlated with genetic variations in angiotensin convertase enzyme (ACE) and transmembrane protease serine 2 (TMPRSS-2). The increase in the spread of the infection could be the result of factors such as geographic diversions and ethnicity. The interaction of the virus with the immune system affects how SARS-CoV-2 appears and spreads [13]. The human leukocyte antigen (HLA)-encoded major histocompatibility complex is responsible for exposing immune cells to viral peptides. Genetic variations in HLA have been shown to control the immune response in SARS infection [12]. The majority of people around the world have already received COVID-19 vaccination, and there have been reports of vaccination altering the immunological response [12,13]. Therefore, there is a need for a trial and study based on the *Prakriti *type and immune response, and such approaches would be helpful in creating new vaccines against COVID-19 new strains [12,14,15]. AG provides genetic expression and an understanding of the effect of phytochemicals on the balance between human health and disorders. Owing to their lower toxicity and side effects compared to manufactured medications and the fact that phytochemical compounds support complicated physiological pathways, plants have long been considered effective sources of medical therapies [14,15,16].

Nearly two-thirds of the world’s population uses plant-based nutritional supplements for health benefits. In addition, they have comparable roles in modern pharmacological biology. Numerous herbs have been developed for their activities against cancer, dental infections, dermatological diseases, diabetes, and anemia [8]. Recently, several Ayurvedic antiviral medications have been employed in COVID-19 patients instead of conventional treatment [17]. Plant micro-ribonucleic acid (miRNA) is a recently identified endogenous short ribonucleic acid (RNA) that negatively regulates a specific messenger RNA (mRNA) and is non-coding. However, by specifically targeting the 3’ untranslated region (3’UTR) of mRNA, certain protein-coding genes are suppressed at the post-transcriptional stage, resulting in mRNA breakage and decreased protein translation. Functional studies conducted in vitro and in vivo have revealed that mammalian miRNAs can block gene expression in helium, marking the first instance of cross-kingdom regulation. This demonstrates that the mammalian digestive tract can target mammalian genes by absorbing plant miRNAs. Hence, the pandemic can be avoided with the use of stratification of genetic differences, variable immune responses, and clinical aspects of the viruses depending on AG and *Prakriti *principles [8].

To effectively manage COVID-19, it is necessary to develop markers that can identify high-risk individuals, recognize the causes of poor prognosis, and understand the variations in therapeutic efficacy. It has been proposed that age, sex, and ethnicity are associated with the prognosis and complexity of COVID-19 [18]. These variances demand an AG-like approach that is not based on race or ethnicity and may make it difficult to identify COVID-19-susceptible groups. Another study claimed that, due to their physiological adaptations, people living at high altitudes are protected from the harmful effects of COVID-19. Studies utilizing the AG method have connected the EGL-9 family hypoxia-inducible factor 1 (EGLN-1) polymorphism, *Prakriti* phenotypes, high-altitude adaption, and susceptibility to high-altitude pulmonary edema (HAPE). This suggests that those with K *Prakriti* may be at a higher risk of developing HAPE, whereas those with P *Prakriti* may be better protected [7,19]. To find an efficient and secure COVID-19 treatment, significant efforts are still being made in the area of medication repurposing. A recent study documented connections between *Prakriti* and the drug-metabolizing enzyme, Cytochrome P450 2C19 (CYP2C19). According to the findings, K *Prakriti* had the highest level of poor metabolizer genotype, while P *Prakriti* had the most extensive metabolizer genotype. It is necessary to conduct these studies for various pharmacokinetic parameters because they can help with dose determination and the monitoring of the side effects of various COVID-19 treatments. From this study, the author suggests formulating clinically pertinent hypotheses that are investigated through rigorous research. The current situation necessitates research on *Prakriti* types pertaining to the genetic, biochemical, and clinical traits of COVID-19 patients [7].

Ayurveda and other healing philosophies employ the hormone modulator Cissampelospareira L. (Cipa) to treat fevers and reproductive disorders. It has also been reported to block the three dengue serotypes, and research has shown how these serotypes affect certain hormones. According to a previous study, Cipa acts on both protein synthesis and estrogen receptors [20]. Numerous medications positively associated with Cipa have been identified as potential antiviral agents. It has been shown that Cipa can stop the spread of viruses, and its chemicals have been shown to bind particularly to the SARS-CoV-2 virus’ spike protein, RNA-dependent RNA polymerase (RdRp), and 3C-like proteinase. In this study, seven minor compounds with strong trademark resemblance to Cipa were identified, including emetine, anisomycin cycloheximide, homoharringtonine, apicidin, ruxolitinib, and sirolimus. The Vero culture assay showed that the entire plant aqueous extract clearly had antiviral properties, as indicated by a drop in relative viral RNA content of 57% at 100 g/ml when the number of viral particles dropped from 105.9 to 105.6. The alkaloid content in the extract was found to be primarily cissamine and magnoflorine, which target the SARS-CoV-2 protein [9].

HIF-1 also plays a crucial role in cases of infection by promoting bacterial and viral replication [5]. One of the most prominent findings in the current COVID-19 pandemic, brought on by SARS-CoV-2, is how severe lung inflammation and other consequences are as a result of the hypoxic response. In a study, Adhatoda Vasica (AV), an Ayurvedic medication with strong anti-hypoxic properties, has lowered the degree of airway inflammation in asthmatic mice by increasing the hypoxic response that is resistant to treatment. The in vitro loss of mitochondrial morpho-function induced by cellular hypoxia was reversed by the anti-HIF-1 activity of AV. According to previous studies, genes linked to the hypoxia-inflammatory pathway are downregulated [10].

Until now, the focus of research has mostly been on individual herbal preparations, with the main goal being to identify and isolate one active ingredient that a pharmaceutical firm could employ. AG strengthens a new connection between medicinal purposes and medical advancements by imparting a firm scientific knowledge of fundamental ideas and incorporating Ayurveda’s useful preventative strategies in medical science. The limitations of this review were the limited number of studies evaluated and the assessment of only pre-clinical laboratory data; hence, the effectiveness of AG in humans was not assessed thoroughly.

## Conclusions

This rapid review found that the viral structure of the SAR-CoV-2 virus was positively influenced by the use of Cipa and AV extracts. With the use of cross-kingdom information, the human genome is correctly stimulated by medicinal herbs. However, drugs based on a patient’s genotype and phenotypic cross-kingdom would be useful in prognosis prediction and treatment tactics. The pandemic can be avoided through the stratification of genetic differences, variable immune responses, and clinical aspects of the viruses depending on AG and *Prakriti* principles. As this is a fledgling subject, more information on AG is needed to help create individualized recommendations that are beneficial to patient health. A customized, time-tested preventive lifestyle and medicine will enable individuals to engage in self-care while assisting modern medicine in its preventative efforts. AG has been shown to have positive effects; however, only a few studies have been conducted in this field. Further studies are needed to gather more data, which calls for research studies and clinical trials involving human patients.

## References

[1] Dey S, Pahwa P (2014). Prakriti and its associations with metabolism, chronic diseases, and genotypes: possibilities of new born screening and a lifetime of personalized prevention. J Ayurveda Integr Med.

[2] Rotti H, Raval R, Anchan S (2014). Determinants of prakriti, the human constitution types of Indian traditional medicine and its correlation with contemporary science. J Ayurveda Integr Med.

[3] Hankey A (2001). Ayurvedic physiology and etiology: Ayurvedo Amritanaam. The doshas and their functioning in terms of contemporary biology and physical chemistry. J Altern Complement Med.

[4] Tiwari P, Kutum R, Sethi T (2022). Recapitulation of Ayurveda constitution types by machine learning of phenotypic traits. PLoS One.

[5] Wallace RK (2020). Ayurgenomics and modern medicine. Medicina (Kaunas).

[6] Page MJ, McKenzie JE, Bossuyt PM (2021). The PRISMA 2020 statement: an updated guideline for reporting systematic reviews. BMJ.

[7] Bhat V, Borse S, Chavan-Gautam P, Joshi K (2022). Exploring AyuGenomics approach for understanding COVID-19 predisposition and progression. J Ayurveda Integr Med.

[8] Bhadresha K, Patel M, Brahmbhatt J (2022). Ayurgenomics: A brief note on Ayurveda and their cross-kingdom genomics. International Association of Biologicals and Computational Digest.

[9] Haider M, Dholakia D, Panwar A (2021). Transcriptome analysis and connectivity mapping of Cissampelos pareira L. provides molecular links of ESR1 modulation to viral inhibition. Sci Rep.

[10] Gheware A, Dholakia D, Kannan S (2022). Adhatoda Vasica attenuates inflammatory and hypoxic responses in preclinical mouse models: potential for repurposing in COVID-19-like conditions. Respir Res.

[11] Nguyen A, David JK, Maden SK, Wood MA, Weeder BR, Nellore A, Thompson RF (2020). Human leukocyte antigen susceptibility map for severe acute respiratory syndrome coronavirus 2. J Virol.

[12] Wang SF, Chen KH, Chen M (2011). Human-leukocyte antigen class I Cw 1502 and class II DR 0301 genotypes are associated with resistance to severe acute respiratory syndrome (SARS) infection. Viral Immunol.

[13] Yamamoto N, Nishida N, Yamamoto R, Gojobori T, Shimotohno K, Mizokami M, Ariumi Y (2021). Angiotensin-converting enzyme (ACE) 1 gene polymorphism and phenotypic expression of COVID-19 symptoms. Genes (Basel).

[14] Verma S, Abbas M, Verma S (2021). Impact of I/D polymorphism of angiotensin-converting enzyme 1 (ACE1) gene on the severity of COVID-19 patients. Infect Genet Evol.

[15] Senapati S, Kumar S, Singh AK, Banerjee P, Bhagavatula S (2020). Assessment of risk conferred by coding and regulatory variations of TMPRSS2 and CD26 in susceptibility to SARS-CoV-2 infection in human. J Genet.

[16] Said-Al H, Hikal W, Mahmoud A (2017). Biological activity of Moringaperegrina: a review. Am J Food Sci Health.

[17] Gupta PK, Sonewane K, Rajan M (2022). Scientific rationale of Indian AYUSH Ministry advisory for COVID-19 prevention, prophylaxis, and immunomodulation. Adv Tradit Med.

[18] Li X, Marmar T, Xu Q (2020). Predictive indicators of severe COVID-19 independent of comorbidities and advanced age: a nested case-control study. Epidemiol Infect.

[19] Arias-Reyes C, Zubieta-DeUrioste N, Poma-Machicao L (2020). Does the pathogenesis of SARS-CoV-2 virus decrease at high-altitude?. Respir Physiol Neurobiol.

[20] Sood R, Raut R, Tyagi P (2015). Cissampelos pareira Linn: Natural source of potent antiviral activity against all four dengue virus serotypes. PLoS Negl Trop Dis.

